# Realising respiratory microbiomic meta-analyses: time for a standardised framework

**DOI:** 10.1186/s40168-023-01499-w

**Published:** 2023-03-22

**Authors:** David Broderick, Robyn Marsh, David Waite, Naveen Pillarisetti, Anne B. Chang, Michael W. Taylor

**Affiliations:** 1 School of Biological Sciences, University of Auckland, Auckland, New Zealand; 2 Present Address: Faculty of Medical and Health Sciences, University of Auckland, Auckland, New Zealand; 3 Child Health Division, Menzies School of Health Research, Charles Darwin University, Darwin, NT Australia; 4 Starship Children’s Hospital, Auckland, New Zealand; 5 Department of Respiratory and Sleep Medicine, Queensland Children’s Hospital, Brisbane, QLD Australia; 6 Australian Centre for Health Services Innovation, Queensland University of Technology, Brisbane, QLD Australia

## Abstract

In microbiome fields of study, meta-analyses have proven to be a valuable tool for identifying the technical drivers of variation among studies and results of investigations in several diseases, such as those of the gut and sinuses. Meta-analyses also represent a powerful and efficient approach to leverage existing scientific data to both reaffirm existing findings and generate new hypotheses within the field. However, there are currently limited data in other fields, such as the paediatric respiratory tract, where extension of original data becomes even more critical due to samples often being difficult to obtain and process for a range of both technical and ethical reasons. Performing such analyses in an evolving field comes with challenges related to data accessibility and heterogeneity. This is particularly the case in paediatric respiratory microbiomics — a field in which best microbiome-related practices are not yet firmly established, clinical heterogeneity abounds and ethical challenges can complicate sharing of patient data. Having recently conducted a large-scale, individual participant data meta-analysis of the paediatric respiratory microbiota (*n* = 2624 children from 20 studies), we discuss here some of the unique barriers facing these studies and open and invite a dialogue towards future opportunities.

Video Abstract

**Supplementary Information:**

The online version contains supplementary material available at 10.1186/s40168-023-01499-w.

## Introduction

Meta-analyses represent a powerful approach to maximise the use of existing data to improve an area of scientific understanding [1]. Such studies often extend the usefulness of data beyond the original research question for which they were generated, allowing the data to continue to provide novel and valuable insights. By combining data from multiple studies, one can dramatically increase statistical power and seek to identify overarching patterns that may not be evident from a single study. This is particularly so for sample types that are difficult to obtain, and therefore, sample numbers are constrained. One such environment is the paediatric lower airways where large-scale microbiota data are limited compared to other fields (e.g. the gut). Individual participant data (IPD) meta-analyses, which utilise more granular data, are even more powerful than meta-analysis of studies where only overall results are considered. As a form of secondary analysis, meta-analyses also allow for the reproduction of original results, a concern highlighted in recently published literature [2, 3]. Additionally, combining data in meta-analyses allows for broader overarching questions — such as determining if there are non-specific disease signatures in the respiratory microbiota — to be investigated in a manner that is largely unfeasible within a single study. Meta-analyses have been proven valuable in furthering understanding of the human microbiota, shining a light on microbiota evolution [4], technical drivers of variation in microbiota studies [5] and providing valuable insights into human disease in both the gut [6] and sinuses [7].

Although often regarded as the highest level of evidence in clinical research [8], the utility of meta-analyses becomes limited where the underlying data are complicated by clinical, technical and/or analytic heterogeneity among the primary studies [9, 10]. Meta-analysis guidelines have been established to address such limitations [11]; however, existing frameworks can be ill-suited to microbiomic datasets that have higher complexity. Standardised frameworks specifying minimum reporting criteria for microbiome studies have emerged (e.g. MIxS [12], STORMS [13] and STROBE metagenomics [14]); however, such frameworks are not yet widely or consistently applied [15], and their suitability for meta-analyses remains to be determined. Context-specific refinement of current frameworks is also needed for respiratory microbiome studies to address heterogeneity related to diagnostic inconsistencies and to account for the different methods used to sample the airways, each of which carries its own biases [16]. Given the complexity and ever-expanding nature of datasets emerging from omics-based studies, the emergence of new airway sampling methods (e.g. breath) [17] and the growing recognition of clinically relevant disease endotypes [18], there is a pressing need to define standardised reporting criteria for respiratory microbiome and multi-omic studies.

Having recently conducted a large-scale, 16S rRNA gene-based IPD meta-analysis focused on the paediatric respiratory microbiota that involved 2624 children from 20 studies [19], we identified several challenges around both data sharing and methodological heterogeneity within this research niche. Here, we examine limitations of respiratory microbiota datasets that emerged from our study [12] and propose strategies to achieve the higher inter-study standardisation needed to support future large-scale respiratory microbiome meta-analyses.

## Data accessibility challenges

After determining a research question, the first challenge in performing any meta-analysis is obtaining access to usable data [20]. Access to published data varies greatly among studies. For our meta-analysis [19], just over half of the 20 included studies had publicly available sequence data, and, at the time of the initial literature search, only two contained sufficient metadata to enable meaningful linking with the sequence data. There was also variability in terms of the types of samples that had been uploaded to public repositories: some publicly uploaded datasets included samples which were not explained or mentioned in the original article, potentially representing mock communities, sequencing controls, negative extraction controls, samples excluded from the original publication or samples from different studies that were uploaded under the same accession number. Key clinical metadata characteristics were frequently excluded, including the anatomical site from which the sample was taken, respiratory diagnosis of the individual providing the sample, and demographic variables such as age and sex, all of which are specified as minimum reporting criteria in multiple reporting frameworks [21, 22]. While contact with corresponding authors yielded access to the required metadata for all the 20 included studies, there is no guarantee that this would be the case for future investigations, with at least one potential study excluded due to a lack of author response. Similar challenges have been encountered in other contexts, including meta-analyses of the gut microbiome [20]. Heterogeneity in the uploading of sequence data to public repositories is also recognised as a systemic challenge in microbiomics that likely stems from the limited training of users who have variable levels of computational experience [15]. There remains a need for best-practice solutions and training to support uploading of sequence data and the associated metadata in consistent and useable formats, as highlighted by earlier standardisation efforts (e.g. MIGS) and exemplified by initiatives like the American National Microbiome Data Collaborative (https://microbiomedata.org/) [15, 21]. As there are currently no applicable metadata reporting criteria for respiratory contexts, we outline the key issues arising from our study, potential solutions and our recommendations in Table 1.

**Table 1:** Issues and potential solutions to data cohesiveness in respiratory microbiota studies

**Issue**		**Short-term solution**	**Long-term solution**
Data accessibility	Ethical challenges	Consider deposition of data in original research proposals	Develop and support the use of guarded archives with established regulatory oversight. Develop patient communication plans that could be used when consenting individuals in order to inform permissions for future data use
	What to upload?	Raw sequence data Clearly labelled controls Minimal metadata^a^	
Cohort heterogeneity	Diagnostics	Clearly state diagnostic criteria for cases Where possible, report symptoms or tests used for decision-making	Determine how accurately current diagnostic criteria reflect pathobiological processes and determine if any can be combined from a microbiota perspective
	Demographics	Report key demographic variables^b^ Try to recruit diverse cohorts	Further research is needed to determine key demographic variables that may confound meta-analyses
	Antibiotics	Create a standardised (and granular) reporting metric for reporting recent antibiotic exposure	Improve understanding of respiratory microbiota recovery from antibiotics
Sample collection	Anatomical site	Address knowledge gaps in lower airway sample literature and (where possible) collect paired upper- and lower-airway samples	Improve understanding as to how related the microbiotas of different respiratory anatomical sites are in different conditions or clinical contexts (e.g. during long-term antibiotic therapy)
	Sampling method	Clearly state sample method used	Determine consistency of different sampling approaches used for the same anatomical site (e.g. nasal swabs and nasal aspirates; sputum and induced sputum; BAL vs protected brushing)
	Sample contamination	Include and report results of negative extraction controls and methods used to mitigate the presence of kitome among low bacterial density samples	
Sample processing	DNA extraction	Laboratory protocols should be reported consistent with STORMS and STROBE-metagenomics requirements [11, 12]	Determine the effects of different extraction methods on perceived microbiota profiles for different anatomical sites
	PCR bias in amplicon sequencing studies		Determine the effects of different sequence regions on perceived microbiota profiles
Bioinformatics processing		Clearly state software, version numbers, and where possible include analysis scripts as supplementary files when submitting primary data for publication	Make data available for secondary analyses which may use a new pipeline

^a^Minimal metadata, diagnosis, age, gender and anatomical site sampled. ^b^Key demographics, age, gender and ethnicity

## Overcoming barriers to accessing primary datasets

The recognised need for improved data-sharing processes has prompted development of frameworks that define current best practices. One recent example is the American National Institutes of Health’s (NIH) Data Management and Sharing Policy that requires data handling according to FAIR (Findable, Accessible, Interoperable and Reusable) principles [23]. While such initiatives will undoubtedly improve data-sharing practices, current frameworks are not specifically designed to address the data access and heterogeneity issues affecting microbiomic meta-analyses. Additionally, time frames for data sharing are not specified beyond being made available “as soon as possible”. Delays in accessing primary datasets, particularly those generated from human cohorts, are a widely acknowledged barrier to secondary analyses [20]. For our meta-analysis, it took ~18 months to access data from the 21 studies that were ultimately included (one of which was excluded following quality filtering of sequence data), while despite best efforts we were unable to access data for a further 10 studies that had met our inclusion criteria. There is broad consensus in the environmental microbiomic field that (sequence and meta)-data should be made publicly available as a condition of manuscript publication [24], thereby removing access barriers entirely; however, such an approach ignores the complex ethical considerations involved in sharing human datasets, particularly those derived from vulnerable or underserved populations [25, 26]. Two such groups are commonly included in paediatric respiratory microbiota studies: firstly, children are an inherently vulnerable population, whose original consent is typically obtained through the proxy of parents, and secondly, marginalised populations who may be overrepresented in respiratory microbiota studies that focus on high disease burdens among vulnerable populations [27–29]. An important example of this is First Nations populations, as emphasised in recent research examining Indigenous data governance frameworks [30]. Informed consent and data privacy are key concerns with respect to data access, as children may wish to remove their data when they reach maturity, a right to be forgotten which has already been acknowledged in other contexts [31, 32]. Reliance on ethics committees alone to determine whether secondary data use is reasonable risks ignoring important considerations related to the rights of First Nations and other vulnerable populations [30]; however, studies of vulnerable populations are essential to improving overall population health, particularly where early interventions may be needed to prevent paediatric conditions progressing into disease in adulthood [33, 34]. Indeed, the importance of diversity within medical research is now increasingly recognised and even demanded [30, 35]. Thus, solving these ethical challenges is imperative to furthering scientific understanding as these issues will only become more complex as datasets become increasingly comprehensive, potentially containing multiple types of microbial sequences (bacterial, fungal or archaeal) as well as human genome data, even if only present through a “bystander” effect in whole-genome shotgun sequencing.

Guarded archives offer a solution for balancing the ethical need to maintain participants’ agency in determining how their data are used against the scientific need for timely access to datasets that are commonly generated using public funds. Guarded archives (databases where sequence and metadata are uploaded but can only be accessed if a request is approved by a recognised data-oversight regulatory body) enable external verification that data presented in original research papers (a) exist and (b) are genuinely accessible to valid research requests (something not necessarily true when data are only made available “upon request” [36]). When paired with data communication plans that are implemented at the time of sample collection, guarded archives provide an opportunity for meta-consent processes to be applied in which individuals can refuse participation in a secondary analysis or withdraw their data from the database at any time. Furthermore, guarded archives allow the validity of new research requests to be reviewed both in the context of the original consent and ongoing patient and community interest group consultation. The Atlas of Living Australia, The Indigenous Background Library and Aotearoa Variome (Genomics Aotearoa, 2019) represent some examples of smaller archives where appropriate Indigenous consultation has been undertaken during development to achieve a more bespoke and ethically sound repository than larger public repositories such as the Sequence Read Archive (SRA) that can be accessed without regulatory oversight. The use of smaller bespoke archives may also mean they are better equipped to deal with the unique challenges facing each individual study in comparison with the monolithic archives currently in common use. Funding and control of bespoke archives, however, bring new challenges as they should aim to be Nagoya Protocol compliant [37], particularly where the microbiota data are later used to develop novel treatments [38]. A limitation of this approach is that smaller organisations managing bespoke repositories may be less able to resist pressure from forensic and law enforcement agencies to release data, a problem already noted with human genome information [39]. It is worth noting that the NIH Data Management and Sharing Policy encourages consideration of controlled access repositories for “sensitive data” [23].

## Heterogeneity in clinical metadata

**Fig. 1:**
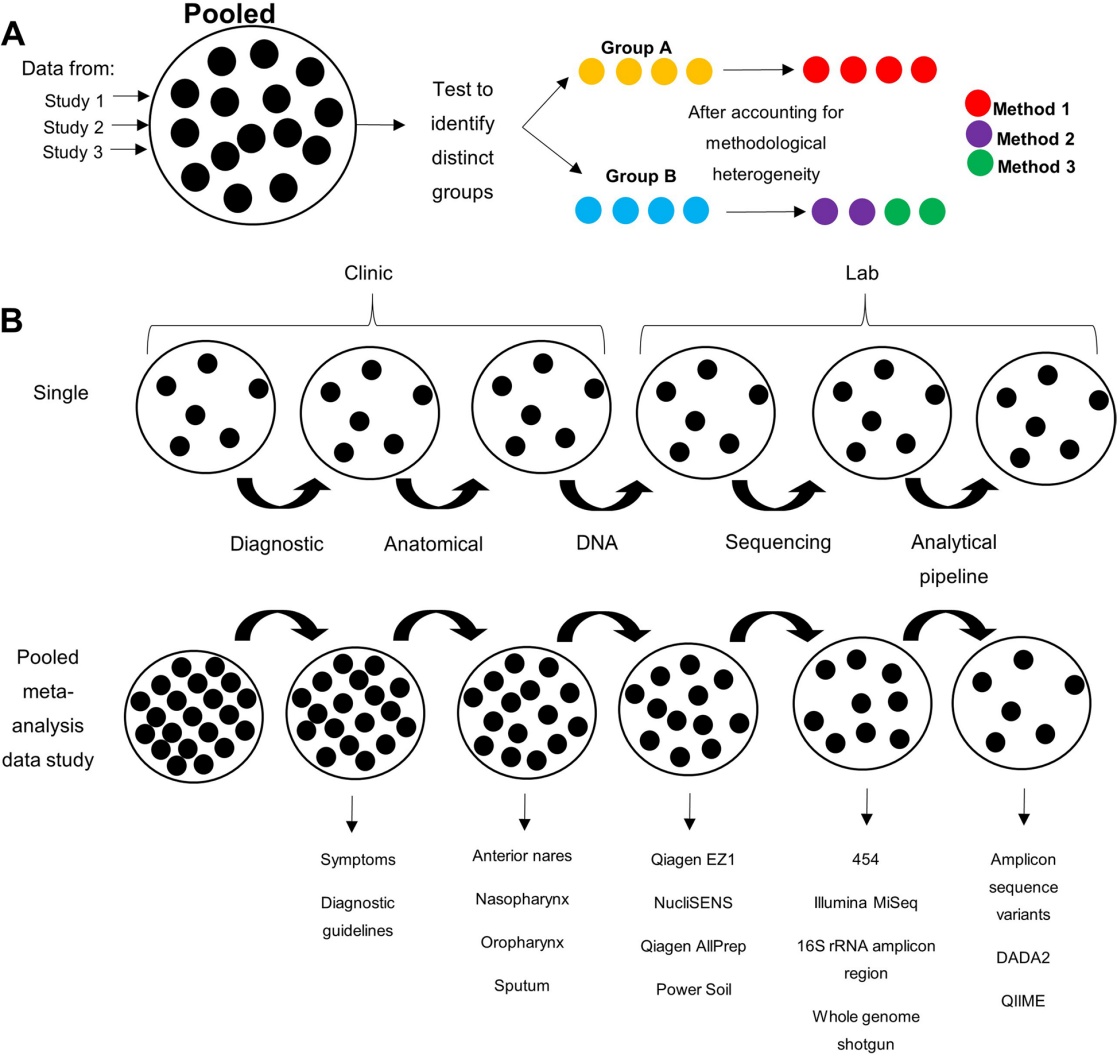
Impacts of data heterogeneity in meta-analyses. A key strength of meta-analyses is the ability to take data from multiple studies to increase sample size. **A** *Methodological heterogeneity risks introducing confounding batch effects in pooled datasets*. In this figure, data from three studies have been pooled and analysed using a uniform pipeline that revealed two distinct clusters (group A and group B). However, after accounting for methodological heterogeneity, it becomes evident that all of the data in group A were generated using a single method that was distinct from those that generated the data in group B. This type of batch effect risks incorrect clinical interpretation of the analysis outputs. **B** *Selecting studies for inclusion in meta-analyses based on standardised methodologies can reduce batch effects outlined in ****A**** but risks loss of sample size*. In respiratory studies, heterogeneity emerges from a multitude of clinic and laboratory factors, from initial differences in diagnostic criteria through to variation in the pipeline used to analyse sequence data. Within a single study (example at top), a given method is applied consistently to all samples and therefore has no impact on sample size. By contrast, within IPD meta-analyses (example at bottom), correction for differences in the methods used at each successive stage progressively erodes the sample size. If the field contains a low level of methodological standardisation, then this reduction could potentially make the statistical power of a meta-analysis no more useful than that of a single study

Another substantial hurdle to IPD meta-analyses, or even “merely” comparing literature on the paediatric respiratory microbiota, is the considerable degree of heterogeneity in study methodology. The sources of this variability stretch from the bedside (where diagnoses, symptoms and treatments may be reported differently) through to sample collection and analytical processing with an overabundance of different laboratory protocols (Fig. 1). Benchmarking protocols for microbiome studies are emerging [40], but, as with reporting guidelines, these remain to be widely adopted. Resolving the variability in the published literature creates a dilemma for meta-analyses, whereby technical differences, which may contribute to inter-study differences, must either be ignored or standardised by data filtration. In our study, this resulted in substantial sample size reduction and therefore constrained our ability to generate findings (Fig. 1).

Assigning a paediatric respiratory diagnosis is a complex process which is influenced by many factors that include country-specific nuances, age of the child, local guidelines and level of expertise such as individual physicians considering factors differently when making clinical guidelines or diagnostic decisions [41, 42]. Even if all diagnostic decisions were consistent, there are limitations as to how much traditional diagnoses may reflect underlying pathobiological processes, for example many diagnoses could contain multiple pathologies with shared symptoms, while common pathologies could present differently in clinic [43]. A recent article highlighted this complexity, calling for the combination and elimination of several diagnoses to clarify this process, aiming to achieve better diagnostic accuracy [43]. Such clarity would also benefit meta-analyses, as diagnostic heterogeneity complicates which patient cohorts should be compared or combined; for instance, microbiota studies of infants have used both respiratory syncytial virus (RSV) infection [44] and bronchiolitis [45] as grouping factors. Despite bronchiolitis being most commonly caused by RSV infection [46], and the high probability that these studies may be discussing identical conditions, the cohorts cannot necessarily be conflated in meta-analyses without additional diagnostic information. Furthermore, while RSV infection in children aged under 2 years is usually clinically diagnosed as bronchiolitis, the same wheezing illness in an older child could be classified as viral-induced wheeze, a non-specific lower respiratory infection. Indeed, the PERCH study identified RSV as the most common cause of pneumonia [47]. Overcoming such challenges requires clearer symptom reporting in microbiomic studies, either at an individual level or by specifying clearly defined case criteria. To address these gaps, we recommend that the criteria in Table 1 be considered as minimum criteria for diagnostic reporting. An alternative approach to a specific clinical diagnosis which is gaining popularity is the concurrent use of endotypes (“distinct molecularly defined functional or pathobiological pathways that may be associated with distinct treatment responses” [48]) and/or phenotypes (clinical parameters of a patient’s condition) or a nuanced approach combining these. Endotypes have proven useful in asthma studies where inflammatory endotypes have been used to successfully identify specific anti-inflammatory therapies [49]. The benefit of using an endotype approach is also reflected in the bronchiectasis field where factors such as sputum production can have high prognostic predictive value [18]. The adoption of respiratory phenotypes and endotypes that are clearly defined and consistent across studies (both in the clinic and in research), rather than a clinical diagnosis per se, will likely be useful in future microbiome-based meta-analyses.

## Accounting for antibiotic usage in respiratory microbiota studies

Respiratory microbiota studies often focus on populations with high antibiotic usage. With antibiotics likely to influence an individual’s microbiota profile [50], consideration of this factor when comparing studies is essential. The STORMS framework requires that authors report data “known about antibiotic usage before or during sample collection” [13]; however, this definition may be too broad in the context of respiratory microbiota studies where different classes of drugs may be used for variable periods of time.

Our meta-analysis [19] identified two broad approaches to reporting antibiotic usage that are common among respiratory microbiota studies: either the time between a patient’s last antibiotic use and sampling is reported or, alternatively, no samples are taken from individuals with antibiotic exposure more recent than a specified cut-off date. The former can be challenging to accommodate in meta-analyses, as a standardised definition of “recent antibiotic use” is lacking. While it is generally believed that the gastrointestinal microbiota will recover in approximately 4 weeks after antibiotic treatment [51], it is unclear whether a comparable window exists for the respiratory microbiota and, if so, how long microbial community recovery might take. Current reporting approaches do provide a way to either standardise or examine the effects of antibiotics within an original study, though comparing across studies with different cut-off dates may mean that antibiotics rather than other biological differences are driving the microbiota differences between study populations. Reporting of patients’ health status at the time of sampling is also important, as the response of the respiratory microbiota to maintenance antibiotics during a period of relative stability and wellness may well be different to that observed during a severe exacerbation where the antibiotic treatment is more intense (e.g. with intravenous antibiotics) [52]. Finally, there remains a need for the effects of different antibiotics to be accounted for in respiratory microbiome analyses. Ideally, more granular data pertaining to the type(s) of antibiotics used, as well as their method of delivery (e.g. oral, inhaled or intravenous), coverage and duration, will be reported. Such data will be essential to achieving a more nuanced understanding of the effects of specific antibiotics than is currently possible when different antibiotic types and classes are analysed collectively under the umbrella of “recent antibiotics”.

## Sample collection in respiratory microbiota studies

The method(s) used to collect respiratory samples is another point of variability among studies that has potential to confound inter-study comparisons and meta-analyses [16]. Despite the respiratory microbiota often being described as a single entity, there are distinct physiochemical niches present in, for instance, the proximal and distal lower airways (e.g. epithelial differences across the bronchi and lung parenchyma) and within the upper airways (oropharynx and nasopharynx) [50]. It remains unclear how similar or different microbial communities may be across the lower and upper airways, with reports indicating both similar [45] and different [53] bacterial communities. It may well be that the connectedness of the communities, and therefore their overall similarity, is differentially altered by illness [54]. A paucity of detailed research on this topic makes it challenging to compare the findings of, say, a study investigating the sputum microbiota with those of a study investigating the nasopharyngeal microbiota, even if the study population has the same condition [55]. Furthermore, within a single anatomical site, there may be differences in the method used to collect the sample. For instance, the microbiota of nasopharyngeal aspirates differs somewhat from nasopharyngeal swabs within the same individual [56]. Similarly, the bronchial microbiota recovered from protected-specimen brushing may be different to that sampled by bronchoalveolar lavage (BAL) or sputum [54]. A nuanced approach to this issue is needed in respiratory microbiome studies, as determining from which anatomical sites samples should be collected, and how this should be performed, differs depending on clinical accessibility, patient tolerance and ethical considerations. While sampling needs to be tailored to the question at hand and the patient population being studied, standardised reporting frameworks would greatly facilitate inter-study comparisons.

## Sample processing heterogeneity

In addition to the aforementioned lack of consistency in clinical diagnoses and sample collection, there is a panoply of ways in which respiratory microbiota studies can progress from a clinically collected sample to ultimately generating a microbiota profile. This includes differences in DNA extraction protocol and selection of sequencing technique (e.g. amplicon sequencing vs shotgun metagenomics). Most respiratory studies to date (and indeed all that were included in our meta-analysis) have employed amplicon sequencing of the 16S rRNA gene. Sources of heterogeneity among the 20 studies we analysed included the 16S rRNA gene region sequenced, PCR amplification and purification parameters, the sequencing platform used and bioinformatic analytical pipelines. While meta-analyses represent a powerful tool to address the variability in bioinformatic pipelines (by analysing all included data via a uniform pipeline), they cannot easily redress the methodological differences which generated the original sequence data. Realising a framework to address this issue in respiratory studies is critically needed as existing sources of heterogeneity will be compounded by the increasing use of new technologies generating additional omics-data types [7, 57] and the emergence of multi-omic applications. Lack of standardisation in laboratory methods used for microbiota studies is a long-standing problem well known in other areas of microbial ecology research [58, 59], including the human gastrointestinal microbiome [60]. While the best protocol for any given study will always be determined by independent research groups, and there exists an argument that true biological signals should transcend differences in methodological approach, large-scale methodological reviews prompted by this heterogeneity in other areas have provided standardised frameworks on which future work to support meta-analyses could be based [60]. For the respiratory microbiota, there is a relative paucity of such studies, and while approaches used to investigate the gastrointestinal microbiota are often taken wholesale and transferred to the respiratory system, there is a lack of evidence that this is the best approach [61].

## DNA extraction methods in respiratory microbiota studies

The DNA extraction method is possibly the factor that most influences observed microbiota profiles [40, 62]. Consideration of this methodology is important, particularly in respiratory studies given the variable bacterial density across the airways that is markedly lower than that observed for gut samples [63]. Failure to account for low bacterial density risks confounding due to reagent contamination or the “kitome”, as highlighted by placental microbiome studies [64]. DNA extraction methods can also result in substantial batch effects which may mask or imitate biological signals [65, 66]. For example, in a longitudinal study of nose microbiomes among infants, Salter and colleagues demonstrated that an apparent association between the composition of the nose microbiomes and the age of the infants was actually due to a DNA extraction batch effect [65]. The application of batch correlation tools, particularly in the limited examples for the respiratory microbiota where both case and control data are available [67], is also worthy of further consideration.

## Sequence analysis in respiratory microbiota studies

Once DNA is obtained from a sample, many respiratory microbiota studies use the 16S rRNA gene amplicon approach to profile an individual’s microbiota. The different regions of this gene which may be selected can lead to different PCR biases and may alter the accuracy of taxonomic assignment [68]. It is not yet clear which 16S rRNA gene region is best to use for respiratory microbiota studies. An analysis of a respiratory-specific database, eHOMD, indicated that the hypervariable V1–V3 regions may provide the greatest taxonomic resolution [69]; however, the length of this region (~490 bp) introduces new challenges related to the limited overlap of forward and reverse reads that occurs when *DNA* > 460 bp is sequenced using most current technologies. As a result of this limitation, many studies instead target the shorter V3–V4 region (~460 bp) where sequencing is performed using the Illumina MiSeq platform. Sequencing of much longer amplicons, as achievable using PacBio technology, could circumvent this issue but may be cost prohibitive. Avoiding amplicon-based approaches altogether, such as in whole-genome shotgun sequencing [16], offers another alternative, although high amounts of contaminating host DNA can be problematic in analyses of respiratory specimens [70, 71], and it is unclear how or if such data could be fairly compared to the majority of current amplicon-based literature.

## Bacterial quantification, diversity and contamination

Bacterial alpha diversity is often used as an indicator of overall respiratory health [34]; however, reporting alpha diversity without bacterial load information can result in errors due to sample contamination and limits our ability to fully interpret these data. The influence of contamination can be exacerbated in respiratory samples compared to, for example, gut samples, given the relatively low bacterial density in the former [63]. Statistical measures used to estimate alpha diversity may also be prone to biases increased by the common practice of rarefaction [72]. As such, direct quantification of total bacterial density (or, most commonly, 16S rRNA gene copies) and/or individual taxa may be more insightful [73], particularly if used in conjunction with alpha diversity metrics, as both overall bacterial community size and diversity can be considered [74]. While several studies use quantitative PCR (qPCR) to estimate bacterial density [53, 75], the myriad of methodologies and inherent variability present within qPCR [76] make inter-study comparisons of such data particularly challenging, especially given the potentially low bacterial density in paediatric respiratory samples [63]. Much like with amplicon-based taxonomic analysis, it is important that standardised reporting processes are encouraged (e.g. MIQE [77]), potentially incorporating new technologies such as droplet digital PCR, which has less inherent variability than standard real-time qPCR platforms [74, 78].

## Concluding remarks

Ongoing research into the microbiota of the paediatric respiratory tract has already yielded promising findings, such as the role of the respiratory microbiota in respiratory tract development [79] and disease susceptibility [80]. This demonstrates the potential of this research avenue to improve understanding of respiratory disease in children. Despite this promise, there remains many more questions and avenues to explore in this complex topic. By cutting through (some of) the noise, meta-analyses offer a promising way to provide new insights into this area while also addressing the issue of small sample sizes that often affects individual respiratory microbiome studies. Such analyses are particularly effective at identifying patterns and generating hypotheses for future investigation and “ground truthing”. However, variability in how studies are conducted and the ways that data are managed and reported can limit both the ability of meta-analyses to reach new conclusions as well as prevent the field from moving forward as a whole, rather than as several splintered research groups. As the microbiome field continues to move toward standardised methodologies [13, 14, 62, 81], these approaches may not necessarily meet the context-specific needs of respiratory microbiome studies, and as such, this work remains to be done. While some potential solutions to these long-standing and complex challenges are offered in Table 1, this manuscript alone cannot propose a complete solution and functions primarily as an invitation for an open dialogue where solutions can be built upon by the field as a whole.

## Data Availability

Not applicable.
